# Metabolic Potential of Cancer Cells in Context of the Metastatic Cascade

**DOI:** 10.3390/cells9092035

**Published:** 2020-09-05

**Authors:** Mohaned Benzarti, Catherine Delbrouck, Laura Neises, Nicole Kiweler, Johannes Meiser

**Affiliations:** 1 Cancer Metabolism Group, Department of Oncology, Luxembourg Institute of Health, L-1526 Luxembourg, Luxembourg; Mohaned.Benzarti@lih.lu (M.B.); Catherine.Delbrouck@lih.lu (C.D.); Laura.Neises@lih.lu (L.N.); Nicole.Kiweler@lih.lu (N.K.); 2 Faculty of Science, Technology and Medicine, University of Luxembourg, 2 Avenue de l’Université, L-4365 Esch-sur-Alzette, Luxembourg

**Keywords:** metastasis, cancer metabolism, one-carbon metabolism, tumour microenvironment, ROS, hypoxia, biosynthesis, bioenergetics, RedOx balance, metabolic plasticity

## Abstract

The metastatic cascade is a highly plastic and dynamic process dominated by cellular heterogeneity and varying metabolic requirements. During this cascade, the three major metabolic pillars, namely biosynthesis, RedOx balance, and bioenergetics, have variable importance. Biosynthesis has superior significance during the proliferation-dominated steps of primary tumour growth and secondary macrometastasis formation and only minor relevance during the growth-independent processes of invasion and dissemination. Consequently, RedOx homeostasis and bioenergetics emerge as conceivable metabolic key determinants in cancer cells that disseminate from the primary tumour. Within this review, we summarise our current understanding on how cancer cells adjust their metabolism in the context of different microenvironments along the metastatic cascade. With the example of one-carbon metabolism, we establish a conceptual view on how the same metabolic pathway can be exploited in different ways depending on the current cellular needs during metastatic progression.

## 1. Introduction

The term metastasis originates from the Greek words meta, meaning next, and stasis, meaning placement [1] and describes fittingly the metastatic process in which a primary tumour spreads to a secondary organ site. The clinical onset of metastasis formation is the result of a complex multi-step process whereby a specific subpopulation of cancer cells within a primary tumour detaches and disseminates into a non-native environment. Such a progression to metastatic disease is the most common cause of cancer related death. While current five-year survival rates of local disease for most cancer types are above 80%, metastatic disease is generally associated with five-year survival rates below 30% [2].

To date, most traditional anti-neoplastic chemotherapeutics have been specifically designed to target rapidly dividing cells [3]. To illustrate, approved drug classes in classical cancer therapy include alkylating agents, antimetabolites, taxanes, alkaloids, anthracyclines, and camptothecin derivatives, all of which interfere with DNA synthesis and thus restrict cell proliferation [4,5]. Such drugs are not especially adopted to slow or non-proliferating, yet potentially malignant cells that can give rise to metastasis. Of note, cancer cell invasion was shown to be negatively correlated with proliferation [6]. This indicates that the invasive, metastatic cell subpopulation might persist when the primary tumour is eliminated by standard antiproliferative cancer therapy. Indeed, primary cancer cells acquire distinct and potentially targetable genetic and phenotypic alterations that favour metastatic progression to distant organs [7]. Prospectively, the enhanced understanding of such specific phenotypic changes in invasive cancer cells could lead to the expansion of current chemotherapeutic regimens and the design of new therapeutic combination schemes. This could include components that do not only target tumour cell proliferation, but also hinder metastatic progression early on. It is thus somewhat surprising that only a minor fraction (<4%) of publications on cancer therapy in general discusses metastatic prevention therapy [8].

In chapter 2 of this review, we briefly revisit the general concept of metastatic progression and metabolic adaptation. In chapter 3, this concept is exemplified with a specific emphasis on the effects of oxygen and nutrient availability in respect of varying tumour microenvironments (TMEs). In chapter 4, we provide a comprehensive insight on the biochemical potential of metabolic pathways that capacitate tumour cells to coordinate their available carbon units for biosynthesis, RedOx balance, and energy production. The potential of metabolic pathways is explored in detail within the context of one-carbon (1C) metabolism.

## 2. Cancer Cells Acquire Specific Traits That Govern Metastatic Progression

During their journey, metastatic cancer cells are described to acquire four specific hallmarks which signify a cell that traverses the metastatic cascade and are indicative of eventual effective secondary tumour formation. Such specific traits include motility and invasion, the ability to modulate the secondary site or local microenvironments, plasticity and the ability to colonise secondary tissues [9].

Cancer cell invasion is a complex sequential and interrelated process, which is initiated by the migration and local invasion of cells from the primary tumour into the surrounding extracellular matrix (ECM). Subsequently, cells that detached from the primary tumour and invaded through the tissue of origin have to intravasate into the vascular system, survive within the bloodstream, extravasate into a distant organ, adapt to the new microenvironment, and multiply to form macrometastases [9,10]. Throughout the metastatic cascade, cancer cells must respond to continuously changing TME conditions, e.g., oxygen partial pressure (pO_2_), nutrients, and mechanical stress [9]. Only cells that successfully adapt to such varying microenvironments between the tissue of origin, the blood or lymph vessels, and the non-native environment in secondary tissues can effectively metastasise. Hereby, cancer cell plasticity provides a selective advantage to allow effective metastatic dissemination to one or several organs. Congruently, a loss of plasticity has been shown to block the ability to metastasise [11]. Mammalian metabolism is one important mechanism that enables such plasticity.

**Figure 1:**
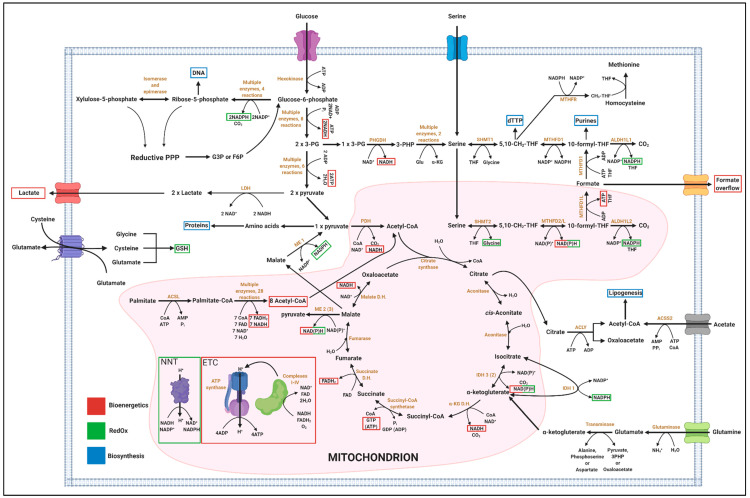
The fate of available carbon sources within cancer cells according to different metabolic sinks. Glucose, serine, glutamine and palmitate are utilised in different pathways to generate co-factors and intermediates that the cell then utilises for biosynthesis (blue), bioenergetics (red) or RedOx balance (green). 3PG, 3-phosphoglycerate; G3P, glyceraldehyde-3-phosphate; F6P, fructose-6-phosphate; PHGDH, phosphoglycerate dehydrogenase; 3-PHP, 3-phosphohydroxypyruvate; Glu, glutamate; α-KG, alpha-ketoglutarate; THF, tetrahydrofolate; SHMT1/2, cytosolic/mitochondrial serine hydroxyl methyl-transferase; MTHFD, 5,10-methylene-tetrahydrofolate dehydrogenase/5,10-methylene-tetrahydrofolate cyclohydrolase; MTHFR, methylene tetrahydrofolate reductase; ALDH1L1/2, cytosolic/mitochondrial 10-formyltetrahydrofolate dehydrogenase; 5,10-CH_2_-THF, 5,10-methylene-THF; CH_3_-THF, 5-methyl-THF; PDH, pyruvate dehydrogenase; LDH, lactate dehydrogenase; ME, malic enzyme; ACLY, ATP-citrate lyase; ACSS2, Acetyl-CoA synthetase 2; ACSL, acyl-CoA synthetase; IDH, isocitrate dehydrogenase; D.H., dehydrogenase; GSH, glutathione.

Metabolic plasticity is not only characteristic to cancer cells but is also a common phenomenon in non-malignant cells. For example, both macrophages and lymphocytes strongly rewire their metabolism upon activation. The way this rewiring takes place depends however on the activating stimulus and cell type and can be directed towards an anabolic or catabolic program [12]. In a similar way, cancer cells are capable of adapting their metabolic phenotype towards their specific needs (Figure 1) [13]. Within a proliferating primary tumour, this rewiring is directed towards an anabolic program to synthesise proteins, lipids, and nucleotides [14]. Throughout the metastatic cascade, the metabolic phenotype can adjust according to current cellular demands during the proliferation-independent dissemination process [6,15].

## 3. Constraints in the Tumour Microenvironment Determine Metabolic Adaptations and Tumour Progression along the Metastatic Cascade

As cells traverse the metastatic cascade, key microenvironmental conditions are constantly altering and may change drastically from solid primary tumour to final macrometastasis establishment in a different organ. In the confined local primary tumour, poor vascularisation has been shown to promote hypoxic regions [16], reactive oxygen species (ROS) stress [17], and altered nutrient availability [18,19,20]. In contrast, disseminated cancer cells may face oxygen-rich environments upon entering the bloodstream or drastically altered nutrient conditions upon seeding in secondary organs [9]. Successful adaptation to these varying conditions can be decisive in the failure or success of metastatic tumour dissemination.

### 3.1. Changing Oxygen and ROS Levels Shape the Cancer Phenotype Along the Metastatic Cascade

Although median oxygen levels in most primary tumours are <2% O_2_, there is significant heterogeneity reported between individual primary tumours in different tissues of origin [21]. Hereby, the highest decrease in pO_2_ in comparison to normal tissue was reported for human pancreatic (19.1–22.7-fold), prostate (2.8–12.5-fold), breast (5.2-fold) and liver cancer (5.0-fold) [21]. Such variability in oxygen saturation between tumour tissues is most probably due to differences in individual tumour vascularisation and the maximal inherent hypoxia tolerance of each tissue of origin [22]. However, somewhat surprisingly, pO_2_ is independent of clinical size, histology and grade for many tumour types [21]. The pO_2_ within tumour tissue can herein be either chronically reduced due to locally prolonged diffusion distances for oxygen within the tissue upon tumour growth (chronic hypoxia) or acutely reduced due to ischemic perfusion limitation in intratumoural blood vessels (acute hypoxia) [21,23]. Signalling of this hypoxic state is hereafter mainly conveyed intracellularly through the accumulation of the hypoxia-inducible factor (HIF)-α subunits HIF-1/2α [24]. Upon hypoxia, HIF-1/2α subunits are stabilised and translocated to the nucleus to form an active transcription factor complex with the constitutively expressed HIF-1β subunit [24]. The subsequent expression of a multitude of HIF target genes in cancer cells has wide-spread promoting effects on angiogenesis, invasion, extravasation, motility, epithelial-mesenchymal transition (EMT) signalling, ECM remodelling, cancer stem cell maintenance, and immune evasion [16,22,23,24,25,26,27,28,29,30,31,32,33,34,35,36,37,38,39]. For example, hypoxia within the primary tumour and the resulting induction of an intra-tumoural hypoxic gene signature were explicitly shown to foster in vivo metastasis formation by promoting a ROS-resistant cancer cell sub-population [40]. Therefore, hypoxic signalling has not only been shown to be characteristic for most established primary tumours, but is also important for their transition to a metastatic phenotype.

Furthermore, HIF-dependent signalling in hypoxic regions of primary tumours is well known to reprogram tumour metabolism. HIF induces glucose metabolism via upregulation of glucose transporters and glycolytic enzymes [41,42,43,44,45,46], increases reductive glutamine metabolism and lipid synthesis [47,48,49] and promotes pentose phosphate pathway (PPP) [50,51,52] to sustain the anabolic phenotype of proliferating tumour cells. One prominent side effect in such highly proliferative, metabolically active cancer cells is the enhanced generation of ROS from increased mitochondrial activity [18,53,54]. On first view, it might be puzzling how hypoxic tumour areas with activated HIF are able to run oxidative phosphorylation (OXPHOS) as this is an oxygen-dependent process. However, it should be noted that oxygen tension usually follows a gradient with a transition zone from oxygen-repleted to oxygen-deprived conditions. Moreover, and maybe even more importantly, OXPHOS can still be active at oxygen concentration of ~1% [55]. Hence, it might be possible that there are transition zones where concomitant HIF activation and active OXPHOS are co-existing. Additionally, there are other factors, which can stabilise HIF, e.g., high ROS level [56]. Interestingly, mitochondrial respiration and ROS derived thereof are important for the adaptation to changing oxygen levels [57]. Enhanced OXPHOS activity has been shown for multiple types of cancers even in the presence of high glycolytic rates [58,59]. This is in contrast to the historic observation of Warburg that led many scientists to assume that upregulated glycolysis in cancer cells would necessarily entail electron transport chain (ETC) downregulation [58]. Rather, cancer cells sustain or even increase their mitochondrial activity and at the same time strongly increase their glycolytic rate. This represents a “full blown” metabolism that works towards its capacity limits to allow fast proliferation [60]. One explanation for increased glycolytic rates is that cells reach their mitochondrial capacity limit. In the case of such limitation, NADH cannot be oxidised anymore via the ETC to regenerate NAD^+^. This can limit the catabolism of nutrients as it requires NAD^+^. Therefore, a way to increase the ATP production rate is to increase glycolysis at the cost of lactate (carbon) overflow. In the case of glucose fermentation, no net production of NADH occurs and the only product that remains in the cell is ATP. When speed and cellular space are the constraints, glycolysis seems to outperform OXPHOS [60,61,62]. This might be pronounced in oxygen transition zones where oxygen availability becomes limiting for OXPHOS.

Upon escape from the primary site and intravasation to blood or lymph vessels, cells are challenged with enhanced oxygen concentrations, which increase from <2% in hypoxic tumour regions to concentrations as high as 4.5% in the lymphatic system and 14% in the blood vasculature [18]. Prominent secondary metastasis sites, such as the lungs, bones, brain, and liver, exhibit oxygen concentrations of up to 4%, 6%, 8%, and 14%, respectively [18]. Thus, progression along the metastatic cascade forces cells to cope with in part drastic increases in oxygen availability and in consequence the enhanced presence of intracellular ROS. Additional sources for ROS in cancer cells encompass the action of oncogenes such as mutant *Ras* and *BCR/ABL* [63,64,65,66], loss-of-function mutations in tumour suppressor genes such as *FH* and *FOXO3a* [67,68], mitochondrial dysfunctions due to mitochondrial DNA mutations of ETC coding genes such as *ND5* [69], inflammatory bursts, and genotoxic stresses upon chemotherapy [53,68,69,70]. The resulting sustained high levels of ROS are termed oxidative stress and are known to exert tumourigenic, cytotoxic, and inflammatory responses as a result of physical damage to lipids, proteins, and DNA. Therefore, effective ROS detoxification is important to prevent cellular damage and emerges as a need for effective metastatic progression [40,51,71]. Indeed, cancer cells often have increased concentrations of glutathione (GSH) and antioxidant defence systems, such as superoxide dismutases, catalases, and glutathione peroxidases, to prevent ROS-induced cell damage [53,72,73,74]. The successful execution of these defence systems depends on increased metabolic activity to supply sufficient GSH to cells and provide sufficient RedOx co-factors in the form of NADPH. NADPH is needed for anabolic processes (i.e., fatty acid synthesis) and concurrently required to provide reducing power to the antioxidant defence system. In the given setting of increased oxidative insults, the available cellular NADPH is mainly required to support ROS detoxification.

The gatekeeper to control cellular ROS homeostasis is the transcription factor NRF2 [74]. Upon ROS-dependent activation, NRF2 induces the transcription of different defence systems including metabolic pathways that support the antioxidant defence [74,75,76]. One important downstream target of NRF2 is the transcription factor ATF4, which activates serine de novo synthesis and 1C metabolism [77]. In the context of ROS defence, serine metabolism is especially required to provide glycine for GSH de novo synthesis. In contrast to excessive ROS levels that are detrimental to cells, it has been shown that low to moderate levels of ROS can trigger pro-migratory and invasive signalling pathways [78,79,80,81]. Thus, being at the right dose, enhanced ROS exposure upon high proliferation rates or in oxygen-rich microenvironments can successfully drive metastatic progression. Exemplary findings include the ROS-dependent induction of cell migration and invasion through the upregulation of ECM-degrading matrix metalloproteases (MMPs) [82], stimulation of MAPK, ERK, and JNK signalling [83], promotion of Rac-dependent actin remodelling [84], and the expression of EMT-associated miRNAs and EMT regulators and marker proteins [85,86]. As such, it is of no surprise that antioxidant treatment has been considered in cancer prevention studies [87,88]. However, recent works by two independent groups have demonstrated that pharmacological and genetic interventions that promote antioxidant programs both resulted in increased metastasis in lung cancer [89,90]. Non-existent beneficial or even adverse effects of such approaches could be explained by the recent finding that ROS effects along the metastatic cascade are highly dynamic. Cheung et al. demonstrated that enhanced ROS levels promote invasion and EMT transition, and, in contrast, reduced survival of secondary metastases in pancreatic ductal adenocarcinoma (PDAC) models [91].

In summary, the shortage of oxygen within hypoxic regions of the primary tumour as well as the enhanced availability of oxygen in lymph and blood vessels and secondary organ sites, have the potential to support cancer progression towards a more malignant phenotype. However, as these oxygen-dependent pro-malignant effects are also on the edge of cell survival, they might be one factor that explains the overall inefficient process of metastasis formation.

### 3.2. The Availability of Nutrients Selects for the Most Metabolically Resilient Cancer Cells

In addition to adaptation to variable oxygen saturation within the different TMEs, the extracellular nutrient availability influences the phenotypic characteristics of cancer cells undergoing metastasis. The TME is thought to be scarce in the availability of nutrients due to the high proliferative activity of the primary tumour and inefficient vascularisation. In a very recent, elegant study, it was demonstrated that in PDAC, nutrient availability in the TME does not reflect what is usually observed from in vitro studies [19]. Even glucose was not fully depleted, however, it was still measured at concentrations lower than those of the plasma [19]. Nevertheless, following on these novel insights, there is common sense that the metabolic signature of the TME is different to healthy tissue and plasma metabolite levels. Yet, the specific metabolic composition is most likely tumour-specific and needs to be considered as context dependent. Given the specific TME, cancer cells will adapt to the local metabolic constraints they are exposed to. For example, disseminating cells, that in contrast to proliferating, biosynthesis-driven cancer cells, do not rely on the de novo synthesis of macromolecules, can engage autophagy to counteract nutrient scarce conditions to better survive the dissemination process [14,92]. Furthermore, it is appreciated that cancer cells often adapt to increased anabolic demands by upregulating nutrient acquisition mechanisms [14]. One example is mutations in molecular switches downstream of growth factor signalling, e.g., PI3K/AKT, resulting in the upregulation of mTOR and the anabolic phenotype [93,94]. Other common mutations are found in the AMPK-activating kinase LKB1 [95], which downregulates the tumour suppressive effects of AMPK. This convergence of different metabolic sensors allows cancer cells to be phenotypically anabolic with no reliance on growth signals [96].

Another advantage gained by tumours in response to stressful nutrient-poor conditions is the selection of clones within the tumour that harbour genetic mutations that offer them survival advantage over other cells in the TME. One example is the selection for cells overexpressing GLUT1 in glucose-reduced conditions [97]. Furthermore, increased expression of pyruvate kinase 2 (PKM2) was shown to promote invasion through regulating MMP2 in lung cancer [98]. Besides glucose, further precursors for lipid, protein and nucleotide synthesis can become exhausted in the TME [99]. For instance, all cells within the TME require lipids in order to synthesise membranes. Breast cancer cells are able to overcome this limitation through acetate uptake and the upregulation of Acetyl-CoA synthetase 2 (ACSS2), which ligates acetate to a CoA group to synthesise acetyl-CoA and hence provides the key precursor for lipogenesis [100]. Another example is seen in about half of melanoma, breast, and lung cancers where tumour cells upregulate PHGDH, the rate-limiting and NAD^+^-dependent enzyme in serine de novo synthesis. Thereby, the cells circumvent a potential dismal depletion of serine in the TME, an amino acid that is essential for protein, lipid and nucleotide synthesis [101,102,103,104]. Interestingly, nutritional intervention to limit serine availability showed anti-tumourigenic effects when PHGDH levels are low [105]. Additional advances in nutritional aspects and cancer metabolism are highlighted in three in-depth recent reviews [106,107,108]. Finally, metabolic waste accumulation can trigger the escape of cancer cells from the primary tumour to metastasise to distal organs in multiple ways. The release of lactate, ammonia, formate, and CO_2_ into the TME from metabolically active cancer cells impacts the pH. This can in turn prompt invasion, e.g., via NFκB, leading to the release of MMP9 and the degradation of the ECM [109,110].

In addition, the TME itself can also support cancer progression. A key aspect of that is sustenance, by which different cell types deposit metabolites that cancer cells use for their proliferation and growth [14]. Glutamate is abundant in the brain as a neurotransmitter and can act as a source for glutamine in cells that harbour the enzyme glutamine synthetase (GS). Glioblastoma cells lacking GS rely on astrocytes to provide them with glutamine, which they require for purine synthesis and hence proliferation [111]. Bone marrow stromal cells support the ability of chronic lymphocytic leukaemia cells to survive elevated levels of ROS through supplying them with the GSH precursor cysteine [112]. PDAC cells are able to circumvent their nutrient-poor TME by relying on alanine deposited by pancreatic stellate cells to fuel their central carbon metabolism [113]. Furthermore, in breast cancer, cancer-associated fibroblasts (CAFs) were shown to be highly glycolytic and can deposit lactate and pyruvate into the TME to support tumour growth [114]. In case lipid supply becomes limited within the adipose tissue in the abdomen, metastatic ovarian cancer cells reprogram CAFs to release cytokines, which in turn enable the cancer cell to metabolise glycogen for energy and biosynthesis [115]. Not only stromal cells of the TME, but also the ECM of the TME impacts tumour development. It was recently shown that the ECM can interact mechanically with cells and inhibit glycolysis. Lung cancer cells are able to bypass such mechanical inhibition through adapting their cytoskeleton and hence maintain high glycolytic rates while other cells in the TME cannot [116]. In general, the composition of the ECM has an impact on the cell’s metabolic phenotype and also impacts the adhesion and migration efficiency of cells [117,118,119,120,121,122].

In order to enter the bloodstream, metastasising cells show traits of EMT, allowing them to escape from the primary tumour and to intravasate [18]. The EMT transcription factor Snail was shown to redirect glycolytic flux towards the PPP to increase NADPH synthesis to balance cancer cell survival during dissemination from the primary tumour [123]. In lung cancer, the depletion of UDP-glucose activates signalling via EGFR and stabilises Snail expression [124]. NFκB signalling in consequence of increased glycolytic activity can upregulate the EMT transcription factors ZEB1/2 [125]. Extracellular lactate was also shown to promote EMT and trigger invasion in renal cell carcinoma through the inhibition of histone deacetylation [126]. The conditionally essential amino acid asparagine was shown to be necessary for EMT gene expression and invasion of breast cancer cells. Knockdown of asparagine synthetase and asparagine starvation both resulted in the inhibition of metastasis without affecting primary lump growth [127]. Very recently, it was also shown that the microbiome impacts the EMT signature and invasive progression of colorectal cancer cells [128] as well as the function of p53 as a tumour suppressor [129].

As detailed in Section 3.1, cancer cells that circulate throughout the body in the bloodstream are exposed to increased ROS levels. However, in contrast to the TME of the primary tumour, glucose is abundant in the bloodstream and thus, these circulating cells are able to utilise this carbon source to balance ROS [123]. A recent study also revealed that the presence of the lactate transporter MCT1 and concomitant lactate consumption positively impacts the metastatic potential of melanoma cells [18,130]. Silencing MCT1 resulted in reduced PPP activity, elevated ROS levels and reduced metastasis formation. This study highlights the high degree of metabolic flexibility of metastasising tumour cells and the need to properly balance ROS during the dissemination stage to reach distant organs [130].

Organotropic metastasis describes the tendency of defined primary tumours to form metastasis in specific secondary organs [131]. While the metabolic niche is not the only factor dictating such relationship, it is indeed an important one. Nutrient availability in the metastatic niche imposes a selection pressure on invading cancer cells and thereby leads to clonal selection of subclones that harbour beneficial metabolic adaptations. The specific nutrient milieu within these metastatic niches and the respective, necessary metabolic adaptations within migrating tumour cells can herein differ significantly depending on the organ primary tumour cells disseminate to. For example, whereas secondary TME conditions in the lungs are dominated by constant oxygen exposure, the liver and brain harbour enhanced concentrations of alternative carbon sources such as acetate, branched chain amino acids and glutamine, that are suited to facilitate high proliferation of specialised metastatic cells [18]. Consequently, primary tumours, which show specific, beneficial metabolic adaptations in heterogeneous cellular subpopulations, are able to form metastases in different secondary organs [18,96]. Evidences of such intricate metabolic patterns of organotropic metastasis have been extensively summarised in specific reviews [18,131,132,133,134]. Targeting OXPHOS in melanoma patients led to a decreased number of brain but not lung metastases [135]. This indicates the existence of at least two clonal subpopulations within melanoma cells that preferentially survive in two distinct metastatic niches and hence indicates a greater chance for cancer progression and therapy resistance [135]. Taken this concept even further, it was recently discovered that in collectively invasive cells, leader cells rely more on mitochondrial activity and PDH activity, while follower cells depend on glucose uptake, confirming that even within one cluster of invasive cells metabolically heterogeneous phenotypes exist [136]. While in Section 2, individual tumour cell plasticity was delineated as an essential trait for successful dissemination throughout the metastatic cascade, these findings additionally highlight the heterogeneity of the tumour and selection of the fittest cell subpopulations as another cornerstone of effective metastatic progression.

In addition to acting as substrates within specific metabolic pathways, metabolites can also have roles beyond their metabolic function. One example for this dual role is the usage of abundant pulmonary pyruvate by metastasising breast cancer cells. On the one hand, pyruvate can anaplerotically re-fill the tricarboxylic acid (TCA) cycle via the activity of pyruvate carboxylase [137,138]. On the other hand, pyruvate can dictate the modification of collagen in the ECM of the lungs to facilitate metastatic growth [139]. Moreover, metabolites can also function as allosteric regulators that impact metabolic fluxes [140] or reshape the epigenetic methylation and acetylation landscape. Impacting epigenetics is feasible as intracellular substrate concentrations to convey such modifications are achievable within the range of the K_m_ values of the respective modifying enzymes [141].

In conclusion, pathways in central carbon metabolism can be dynamically altered to adjust to metabolic constraints during the metastatic cascade. Hereby, engagement of the same central pathways can lead to distinctively different metabolic outputs. For example, cancer cells can utilise glucose as carbon-source for bioenergetics, biosynthesis or RedOx balance generating in the process different and proportionally variable amounts of co-factors, building blocks and ATP. Hereby, cancer cells in the primary tumour are for instance able to adapt their metabolism to proliferation rates that exceed the capacity limit of the ETC [60]. Metastatic tumour cells, on the other hand, have to withstand harsher metabolic environments that require a different re-wiring of their central carbon metabolism to counteract oxidative stress, allow cell survival and effective outgrowth at metastatic sites. The following section of the review will thus explore the metabolic potential of the pathways of central carbon metabolism by looking at their energy and co-factor balances exemplifying how each pathway can be exploited.

## 4. Metabolic Flexibility Permits Adaptation to Changing Microenvironments

In Table 1, Table 2 and Table 3, examples are provided on how substrate utilisation can vary if bioenergetics, biosynthesis, or RedOx demand is predominant. The focus is hereby set on the major cellular carbon substrates glucose, serine, fatty acids (FA; with palmitate as an example) and glutamine [99].

Table 1 summarises how these main energy metabolites can be oxidised to maximise ATP generation and thereby cellular energy production. Glucose molecules traverse glycolysis in the cytoplasm generating two pyruvate, two ATP, and two NADH molecules. One pyruvate molecule can either be reduced to lactate (consuming one NADH molecule in the process) or be fully oxidised in the mitochondrion to generate three CO_2_, one ATP, four NADH, and one FADH_2_ [142]. Serine can be catabolised to produce glycine and formate, which both can be secreted from cells in significant amounts [143]. This catabolism generates one ATP and one NAD(P)H per serine molecule [144]. Of note, using stable-isotope tracing to follow the fate of serine, serine catabolism to pyruvate via serine dehydratase was not observed. Instead, all serine was catabolised to glycine and formate via 1C metabolism [143]. Complete oxidation of FA stoichiometrically yields a number of acetyl-CoA molecules that equates half the number of carbons that the FA contained. Hereby, the generation of each acetyl-CoA molecule is accompanied by the production of one NADH and one FADH_2_ molecule. Subsequently, each acetyl-CoA enters the TCA cycle to be fully oxidised producing two CO_2_, three NADH, one FADH_2_, and one ATP [142]. Glutamine is readily converted to α-ketoglutarate, which passes through the TCA cycle up to malate. Malate is then oxidised to pyruvate through the action of malic enzyme 1, 2, or 3 (ME) producing cytosolic NADPH, mitochondrial NADH or mitochondrial NADPH, respectively [145,146,147]. Subsequently, lactate or acetyl-CoA can be produced from pyruvate. Alternatively, pyruvate can be oxidised by PDH and fed back into the TCA cycle. If 50% of glutamine-derived malate is converted to oxaloacetate, while the other 50% are converted to pyruvate via ME, glutamine can be fully oxidised via the TCA cycle without the need of any other carbon sources. If palmitate and glutamine are both catabolised, glutamine-derived oxaloacetate and palmitate-derived acetyl-coA can be oxidised jointly.

Table 2 summarises the potential metabolic products generated from the above-discussed main metabolic substrates if biosynthesis is the primary demand. As seen in Figure 1, glucose can provide carbon units for every major building block (nucleotides, lipids, and proteins) by supporting glycolysis (glycerol for lipids, 3PG for serine and hence nucleotides, pyruvate for alanine), PPP (riboses for nucleotides) or TCA cycle (acetyl-CoA for FA and different non-essential amino acids for proteins). The cytosolic acetyl-CoA pool is the main source for FA synthesis. This pool is mostly filled through the activity of ACLY (Figure 1), which cleaves cytosolic citrate into acetyl-CoA and oxaloacetate [142]. Moreover, a recent study found that pyruvate can be oxidised to CO_2_ and acetyl-CoA in a ROS-dependent manner [148]. This alternate mechanism could be of relevance during invasion, a process that has been shown to be propagated by ROS as detailed in Section 2 of this review [78,79,80]. Cytosolic citrate levels are mainly sustained via glucose through glycolysis and the TCA cycle or glutamine depending on the cell’s state and TME [47,49,149,150]. However, it is important to keep in mind that biosynthesis is also an energy expensive process that requires significant amounts of ATP to build up macromolecules. This ATP needs to be generated by the cells in parallel to the consumption of available carbons for macromolecule synthesis. As the cell dry mass composition is well defined for mammalian cells [151] and costs for protein translation, lipogenesis and nucleotide synthesis are also known [142], the energy demand for the duplication of the cell’s content can be estimated. Such estimation revealed that the duplication of 1 litre cell volume costs ~6 moles of ATP, which is a high demand of ATP molecules if set into context with e.g., a blood glucose concentration of 5 mmol per litre [60]. In addition to ATP, NADPH is needed for de novo FA synthesis. Such extensive carbon demand for biosynthesis makes it conceivable that the absolute intracellular carbon demand is highest in proliferating cells. This further underlines the concept of a “full blown” metabolism described in chapter 2.

Table 3 provides an overview of metabolic routes when maintenance of ROS balance (RedOx homeostasis) is the predominant cellular constraint. PPP is divided into two parts, namely oxidative and reductive, providing ribose-5-phosphate for nucleotide synthesis and cytosolic NADPH for RedOx balance [142]. Depending on the intracellular and TME conditions, a cancer cell determines whether to run both, the oxidative and reductive part of the PPP or only the oxidative part. During anabolic state and biomass generation, glucose carbons are metabolised exclusively by the oxidative PPP to generate ribose units for biosynthesis. This oxidative path yields 2 mol NADPH per mol glucose. However, if maintenance of RedOx balance is the main interest, carbons are cycled through the oxidative and reductive PPP in parallel to generate the maximum possible number of NADPH from one glucose molecule [142]. Per cycle through the oxidative PPP, the cell loses one carbon atom in form of CO_2_. NADPH is used by cells as a reducing power to reduce the oxidised form of GSH, which is then in turn utilised to reduce ROS and maintain RedOx balance [152]. In consequence, NADPH production will be the major sink of glucose carbons when the cell faces a RedOx imbalance. A recent report nicely demonstrated by non-stationary flux analysis that cells can quickly adjust their glycolytic metabolism towards PPP to maximise NADPH production. Upon oxidative insult, GAPDH was oxidised, which blocked glucose carbons from entering lower glycolysis and redirected these carbons through G6PDH for full oxidation via PPP [153]. Apart from PPP, the other two main sources of cytosolic NADPH are the enzymes ME1 and IDH1 (Figure 1, Table 3) [154,155]. RedOx cycling between cytosolic IDH1 and mitochondrial IDH2 can be used to specifically counteract mitochondrial ROS to support anchorage-independent growth [149,156]. Additionally, mitochondrial NADPH can be potentially generated through the activity of nicotinamide nucleotide transhydrogenase (NNT), which is capable to convert NADH to NADPH [157,158]. This pathway is represented in Table 3 as part of the oxidation of palmitate, which is a major source for mitochondrial NADH.

It should be noted that the purpose of these tables is to illustrate how one pathway can be potentially maximised towards a certain constraint. In reality, different intermediate combinations will exist depending on the cellular and microenvironmental context. This concept of metabolic flexibility and adaptation occurs in the cell as one complex system. For the sake of simplicity, it is possible to zoom into one metabolic pathway and dissect its potential with respect to different metabolic sinks. Chapter 4 of this review will take an in-depth look into 1C metabolism and exemplarily highlight its potential in supporting each of the above-discussed constraints.

### 4.1. One-Carbon Metabolism has Implication in Biosynthesis, Bioenergetics and RedOx Control

**Figure 2:**
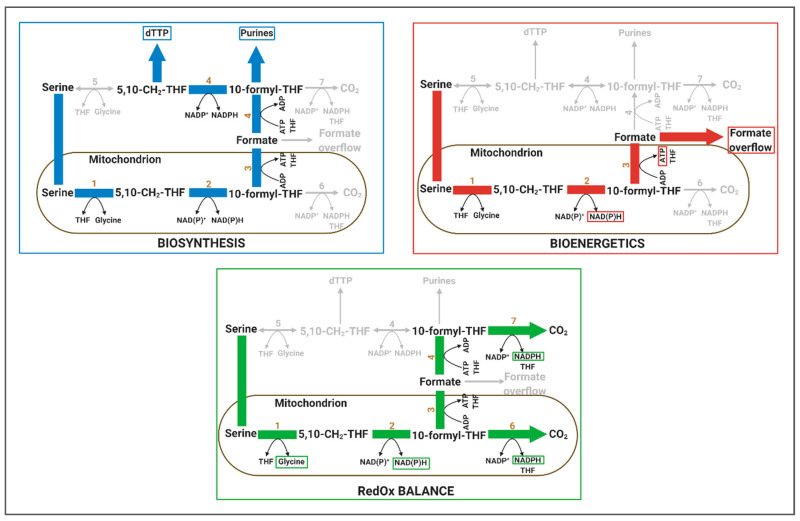
One-carbon cycle and its theoretical potential in supporting biosynthesis, bioenergetics and RedOx balance. One-carbon (1C) cycle can support biosynthesis through the generation of 10-formyl-THF and 5,10-CH_2_-THF. When formate is released from the 1C cycle, this yields one NADH and one ATP molecule per serine supporting energy balance. Alternatively, the 1C unit can be released as CO_2_ generating NADPH in the process through the action of ALDH1L1/2. NADPH can also be generated from the action of MTHFD2/L. In addition, serine-derived glycine can be used for purine synthesis (biosynthesis) or GSH synthesis (RedOx). Enzymes are abbreviated with numbers as following: 1: SHMT2; 2: MTHFD2/L; 3: MTHFD1L; 4: MTHFD1; 5: SHMT1; 6: ALDH1L2; 7: ALDH1L1. 5,10-CH_2_-THF, 5,10-methylene-THF; THF, tetrahydrofolate; SHMT, serine hydroxyl methyl-transferase; MTHFD, 5,10-methylene-tetrahydrofolate dehydrogenase/5,10-methylene-tetrahydrofolate cyclohydrolase; ALDH1L, 10-formyltetrahydrofolate dehydrogenase.

Folate-mediated 1C metabolism describes a series of biochemical reactions that governs the transfer of 1C units from donor metabolites to different acceptors (Figure 1 and Figure 2) [144,159,160]. Herby, the main donor metabolite is the non-essential amino acid serine. In contrast to the TCA cycle, which is exclusively located to the mitochondrion, the 1C cycle spreads across mitochondrion and cytoplasm. In the first, mitochondrial part of the cycle, serine is catabolised to glycine and a 1C unit (either formate or CO_2_) under production of reducing equivalents and ATP. In the second cytoplasmic part of the cycle, both glycine and formate can subsequently serve as precursors for anabolic reactions or serine can be re-synthesised which then closes the 1C cycle. Thus, 1C metabolism is an example of a pathway in which catabolic and anabolic reactions are separated from each other by cellular compartmentalisation.

Of note, serine catabolism via the 1C cycle is also connected to the methionine cycle by donating CH_3_-THF for the remethylation of homocysteine to methionine (Figure 1) [144]. However, the quantitative contribution of 1C units to the methionine cycle is in most cases rather low and environmental methionine seems to be critical in most tissues [159,161]. Therefore, we do not include the methionine cycle in our discussions in this section.

#### 4.1.1. Relevance of 1C Metabolism in Biosynthesis

1C metabolism became a hotspot in cancer research due to its essential role in the de novo synthesis of nucleotides, which renders it crucial for biosynthesis in proliferating cells [162]. To synthesise a purine molecule, one glycine and two 1C units are required. For the synthesis of one dTMP, one 1C unit is required. As purine demand in mammalian cells strongly exceeds pyrimidine demand, the absolute purine flux is higher than the 1C flux for dTMP synthesis. dTMP is only required for DNA synthesis and the cellular ratio of RNA to DNA is roughly 3:1 [163]. The highest demand is posed by the synthesis of adenine, as this purine is not only needed as a building block for DNA and RNA, but also for cellular ATP, which is present in millimolar concentrations. Inhibition of serine catabolism can inhibit proliferation by blocking nucleotide synthesis. However, when only mitochondrial serine catabolism via SHMT2 is inhibited, a cytosolic analogue of the enzyme, SHMT1, can compensate to a certain extend and sustain growth [164]. In addition to nucleotide synthesis, glycine is required for haem and GSH biosynthesis. Besides ATP, GSH is one of the few other metabolites that are concentrated within the millimolar range. Therefore, GSH represents a major sink for glycine. Targeting GSH de novo synthesis via the rate-limiting enzyme glutamate cysteine ligase has been shown to have detrimental effects in cancer cells as well as in immune cells [74,165]. Finally, serine is a proteinogenic amino acid that is also consumed for protein biosynthesis.

#### 4.1.2. Relevance of 1C Metabolism in Bioenergetics

To generate formate, the 1C unit CH_2_-THF is oxidised via MTHFD2 which in turn reduces NAD(P)^+^ to NAD(P)H, a RedOx-relevant reaction. As oxidation of CH_2_-THF is located in the mitochondrion, the generated NADH can readily be oxidised at complex I of the ETC, which generates 2.5 mol ATP per mol NADH. It is under debate if the oxidation of CH_2_-THF in cancer cells is coupled to NAD^+^ or NADP^+^. Early in vitro results pointed towards NAD^+^ [166], while later the Appling lab provided evidence by biochemical in vitro assays that MTHFD2 shows similar kinetics for both co-factors to oxidise polyglutamated CH_2_-THF [167]. Yet, a study investigating this question in intact cells remains to be undertaken. However, the different concentrations of the co-factors in the mitochondrion and the experimental evidence that inhibition of the ETC, which is required to oxidise NADH, inhibits mitochondrial 1C unit oxidation [143,168] indicate that, in intact cells, the oxidation of CH_2_-THF depends on NAD^+^ and active respiration. This is supported by a recent study showing that MTHFD2 can still oxidise 1C units at decreasing NAD^+^/NADH levels at which enzymes of the TCA cycle are already inhibited [169]. In such case, a larger fraction of OXPHOS-derived ATP could originate from serine catabolism. It therefore implies that mitochondrial 1C metabolism might have a meaningful role in bioenergetics under specific TME settings. Yet, it is important to note that even if MTHFD2 is less sensitive to feedback inhibition by NADH, the absolute flux of 1C unit oxidation is strongly inhibited when the ETC is strongly inhibited (e.g., by severe hypoxia or complex I inhibition) [143,168,169,170]. Thus, even if the relative flux might not change, the absolute flux clearly decreases when the respiration is inhibited. This is best illustrated by the observation that serine consumption rates and formate release rates strongly decrease when the ETC is blocked [135,161]. Hence, the quantitative analysis of serine consumption and formate release rates provides a robust tool to measure the rate of serine catabolism.

Once formyl-THF (CHO-THF) has been generated from CH_2_-THF by the activity of MTHFD2, formate can either be further oxidised by ALDH1L2 to CO_2_ under production of NADPH or released from THF under production of ATP. Hence, depending on the environmental condition, the fate of 1C units can differ and the catabolism of serine can support RedOx and/or bioenergetics. Under standard cell culture conditions, it was observed that in proliferating cells the largest fraction of generated 1C units are excreted in the form of formate in a process designated as formate overflow despite the cell’s high demand of 1C units for nucleotide synthesis [143]. In a subsequent study, this phenomenon was also observed in different oxidative tumours in vivo [170]. Release of every formate molecule indicates a net production of ATP at the price of carbon loss that could otherwise be used for biomass production. Formate overflow has therefore some parallels to lactate overflow. In both cases, all carbon is lost and ATP is gained. However, the difference is that lactate overflow is cytoplasmic and formate overflow depends on the mitochondrial activity. Using different cancer models, it was observed that under nutrient stress conditions, when glycolysis is limited and cell growth is arrested, serine catabolism and formate overflow increases [143]. Whether or not this is mediated via AMPK, which was also activated under these nutrient stress conditions, remains to be elucidated. Although the detailed physiological relevance still needs to be unravelled, these observations suggest an additional catabolic role for serine metabolism via the 1C cycle. First results demonstrated that serine catabolism and its product formate can promote invasion of glioblastoma cells, a growth-independent process that could promote metastatic progression [170].

In summary, the overall balance of mitochondrial serine catabolism is:

1 serine + 1NAD^+^ + 1ADP → 1 glycine + 1 formate + 1ATP +1 NADH (1)

In the case of NADH oxidation via the ETC and formate excretion, the catabolism of serine generates 3.5 ATP with only four enzymatic reactions, which equals 0.88 mol ATP per reaction (Table 1). This is a similar efficiency as full oxidation of glucose to CO_2_ via glycolysis and TCA cycle. Compared to glutamine oxidation, serine catabolism has also a reduced NADH burden, which could be of advantage in cancer cells that are likely to struggle to regenerate sufficient NAD^+^. Thus, in growth-independent conditions or glucose-deprived TME, catabolism of serine could be an attractive pathway to exploit serine as an energy substrate. Alternatively, if, e.g., the level of oxidative stress and therefore the demand for reducing equivalents rises, formate could instead be directed to ALDH1L2 for full oxidation and concomitant NADPH production [51,154].

#### 4.1.3. Relevance of 1C Metabolism in RedOx

If formate is not excreted from the cytoplasm, it can be bound again to THF under consumption of 1 ATP, which renders the ATP balance of the 1C cycle zero. Cytosolic CHO-THF can then be used for purine synthesis or alternatively be reduced to CH_2_-THF by cytosolic MTHFD1. CH_2_-THF is required for the synthesis of the pyrimidine thymine and it has been shown that most CH_2_-THF for dTMP synthesis is derived from the mitochondrion [171]. Interestingly, MTHFD1 is strictly NADP^+^-dependent [144]. Thus, regeneration of CH_2_-THF in the cytoplasm results in net consumption of NADPH. Experimental evidence showing that a significant fraction of serine-derived 1C units are resynthesized to serine in the cytoplasm indicates that 1C cycle consumes NADPH [164]. Following this logic, it appears more rational to fully oxidise formate to CO_2_ by ALDH1L2 or ALDH1L1, if maintenance of the RedOx balance is the major cellular constraint (Figure 1 and Figure 2) as in that case, there will be net production of NADPH by 1C cycle. In fact, a role for ALDH1L2 in RedOx control has recently been reported. When cells were put under nutrient stress, and hence exposed to increased ROS, ALDH1L2 activity increased to produce NADPH [154].

Given these biochemical relations, it nevertheless remains debatable if 1C metabolism represents a general major source for cellular NADPH. At first, in a pioneering study, 1C metabolism got into the spotlight for being a major contributor to cellular NADPH metabolism [172]. However, by showing that the co-deletion of ME1, IDH1, and G6PDH was detrimental to cancer cells and that no other pathway could compensate for the resulting lack of NADPH, the same lab was able to demonstrate in an elegant way that 1C metabolism has no major role for cytosolic NADPH production [155].

Even if not critical for the cytosolic NADPH pool, 1C metabolism could be of relevance for NADPH homeostasis in the mitochondrion under certain environmental conditions as has been shown previously [154]. However, in that case, NADPH is most likely derived from the activity of ALDH1L2 rather than MTHFD2 [164]. Yet, a quantitative comparison of NADPH production from ALDH1L2 compared to mitochondrial IDH2 and ME3 to elucidate the respective fractional contributions to the mitochondrial NADPH pool still needs to be conducted.

#### 4.1.4. Systemic and Organ-Specific Aspects of 1C Metabolism

1C metabolism has also a significance on a systemic level. In a nutshell, most oxidative organs show glycine and formate overflow, while the kidney and especially the liver are thought to be glycine and formate sinks and to re-synthesise serine [160,170,173]. This whole-body cycle is important to keep the plasma levels of the 1C metabolism-related metabolites constant. To allow efficient recycling, liver and kidney express higher levels of MTHFD1 than most other organs. Therefore, using kidney or liver cell lines can lead to organ specific results that might not fully reflect other tissue-derived cancer cell lines [164]. In the landmark paper of Fan et al., quantitative analysis of 1C metabolism contribution to the cellular NADPH pool has been performed in immortalized baby mouse kidney cells (iBMK cells) [172]. In a follow-up publication of the same lab it was also demonstrated by using deuterated serine tracers that iBMKs show an unusual high activity of cytosolic serine catabolism via SHMT1 [164]. Here, it was concluded that mitochondrial 1C cycle-derived NADPH is mainly produced from the activity of ALDH1L2 [164]. Despite initial confusion around the role of 1C metabolism in cellular NADPH homeostasis, several excellent follow-up studies of the same lab as well as many other labs, advanced our understanding of 1C metabolism tremendously over the last five years.

By in-depth discussion of the example of 1C metabolism flexibility, it was illustrated how a single metabolic pathway can play different roles in bioenergetics, biosynthesis and RedOx homeostasis. It highlights thereby that cellular metabolism has the potential to adapt to different constraints within the TME. The observation that nutrient availability can crucially alter the directionality and output of a plethora of metabolic pathways shows how vital the microenvironment is in dictating metabolic rewiring.

## 5. Concluding Remarks: Differential Carbon Distribution Allows Cells to Survive the Metastatic Cascade

**Figure 3:**
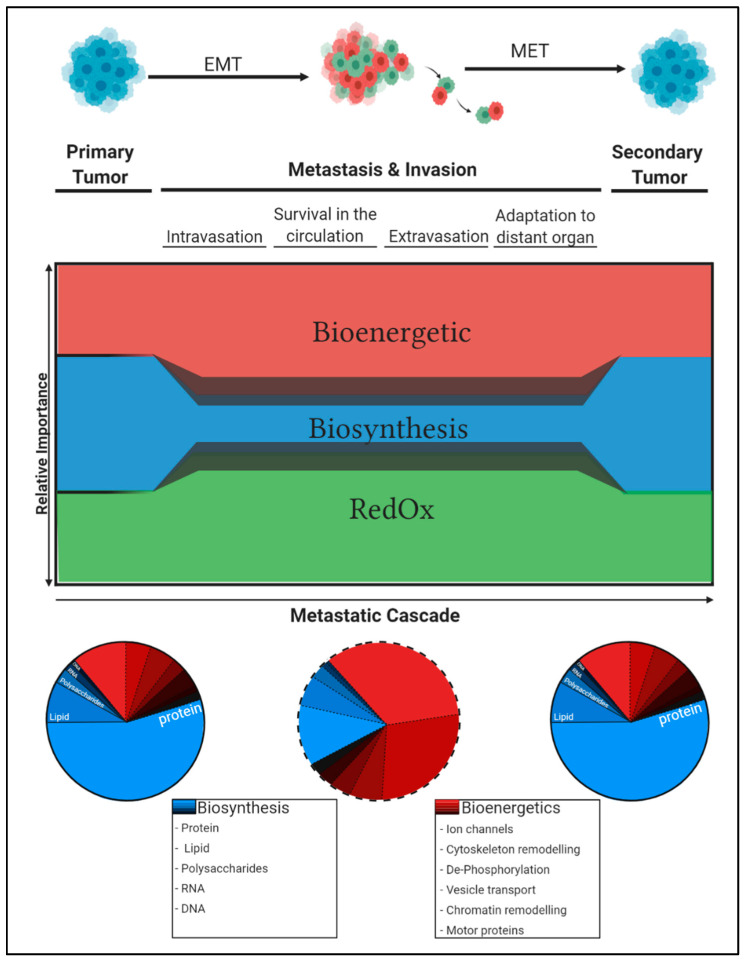
Flexible metabolic conversion of available carbon sources allows cancer cells to adapt to changing metabolic needs along the metastatic cascade. According to the cellular needs, the available carbon atoms from nutrients are distributed between the three main metabolic sinks. The relative amount of carbon atoms that is invested for proliferation in primary tumour cells prohibits extensive energy allocation for invasion and metastasis. However, under growth-arrested conditions, cells spend less nutrients on biosynthesis and can reallocate the gained spare carbons to promote an invasive phenotype by investing in enhanced cellular energy production and/or improved anti-oxidant defence. Thus, upon proliferation within the primary tumour and in the developed metastasis (left and right pie chart), the cells invest most energy for building block production (protein, lipid, DNA/RNA synthesis). However, during the metastatic process (middle pie chart), the metastatic cells switch from anabolic to catabolic metabolism and energy might be redistributed to support cell invasion.

It appears that, during dissemination, nutrient catabolism is not used for proliferation but instead can be exploited for survival and motility. During metastasis, cells are in cell cycle phase G0/G1 [6] and consequently, the energy demand to synthesise macromolecules is low. Under such growth-independent conditions, the suitable cellular adaptation by targeted rewiring of metabolic pathways can support cells with spare carbon units to be used for maintenance of RedOx balance and the generation of energy. Both of these carbon sinks are imperative to establish and support a motile invasive phenotype (Figure 3) [119]. Such spare energy and residual RedOx potential might be used during metastasis for “mechanical work” and the counteraction of oxidative stress (Figure 3).

We are just now starting to understand how the detailed metabolic adaptations towards these metastatic constraints and demands look like. However, we currently lack sufficient in-depth knowledge and quantitative insight on these adaptation processes. Being able to comprehensively understand the dimensionality of metabolic adaptations and the intricate ways they manifest will allow for the design of tailored intervention strategies directed specifically against more dormant, metastasis-prone tumour cells. As potential interventions can vary from patient to patient, precision medicine and appendant patient-specific analysis of cancer metabolism may open new possibilities to define individual treatment regimens, even though there is still a long way to go to attain this final goal.

## Figures and Tables

**Table 1:** ATP and co-factors produced from glucose, palmitate, serine and glutamine when bioenergetics are the main metabolic constraint. Glucose can either be oxidised to 2 lactate or to 6 CO_2_ molecules. One carbon (1C) metabolism can generate 1 ATP if only the mitochondrial 1C cycle is run and the end-product formate is not recycled. MTHFD2/L has two separate enzyme activities, a dehydrogenase and a hydrolase activity. Complete oxidation of a fatty acid e.g., palmitate yields 16 CO_2_ and generates energy in the form of 6 ATP, 31 NADH and 15 FADH_2_ molecules. Breakdown of glutamine in the TCA cycle and subsequent conversion of malate to pyruvate through the action of malic enzyme 2 (ME2) generates 1 ATP, 1 NADH and 1 FADH_2_ molecule. Total ATP for each reaction is calculated based on ATP molecules generated, each NADH molecule producing 2.5 ATP molecules and FADH_2_ producing 1.5 molecules of ATP molecules upon OXPHOS. * Depending on the shuttle system used to transport cytosolic NADH to the mitochondrion, 2.5 or 1.5 ATP molecules can be produced per molecule cytosolic NADH in this case [142]. Gly, glycine; HCOO^-^, formate; FAO, fatty acid oxidation; TCA, tricarboxylic acid; ME, malic enzyme; RXN, reaction; C, carbons per substrate molecule.

Substrate	Pathway	C	RXNs	Products	ATP	NADH	FADH_2_	Total ATP	ATP/RXN
Glucose	Glycolysis	6	17	2 Lactate	2	0	0	2	0.12
Glucose	Glycolysis TCA cycle	6	35	6 CO_2_	4	10	2	30 or 32 *	0.86 or 0.91
Serine	Mitochondrial 1C Cycle	3	4	1 Gly, 1 HCOO^-^	1	1	0	3.5	0.88
Palmitate	FAO TCA cycle	16	101	16 CO_2_	6	31	15	106	1.05
Glutamine	TCA Cycle ME2	5	7	Pyruvate, 2 CO_2_	1	2	1	7.5	1.1

**Table 2:** ATP and co-factors produced from glucose, serine, and glutamine when biosynthesis is the main metabolic constraint. Metabolism of glucose can downstream contribute to the synthesis of nucleotides, fatty acids, amino acids and ribose-5-phosphate through the utilisation of its carbon atoms within specific pathways. One-carbon cycle is utilised to free one-carbon units from serine to generate purines and dTTP. Glutamine can anaplerotically support the TCA cycle and generate cytosolic citrate through reductive carboxylation which subsequently feeds into fatty acid synthesis. NADH generated per product is shown here to indicate the burden of NADH during anabolic state [60]. 3PG, 3-phosphoglycerate; Gly, glycine; FA, fatty acids; THF, tetrahydrofolate; 5,10-CH_2_-THF, 5,10-methylene-tetrahydrofolate; dTTP, deoxythymdine triphosphate; RXN, reaction; C, carbons per substrate molecule.

Substrate	Macromolecule	C	RXNs	Products	ATP	NADH	NADPH	Total ATP	NADH/Product
Glucose	Nucleotides	6	15	2 serine	−1	4	0	9	2
Glucose (via ACLY)	FA	6	21	2 Acetyl-CoA 2 CO_2_	0	4	0	10	2
Glucose	Amino Acids	6	17	2 Alanine	2	2	0	7	1
Glucose	Ribose Sugar	6	5	Ribose 5-phosphate, CO_2_	−1	0	2	−1	0
2 Serine	Purines	6	10	2 10-formyl-THF, 2 Gly	0	2	0	5	1
Serine	dTTP	3	7	5,10-CH_2_-THF, Gly	0	1	−1	2.5	1
Glutamine (reductive route)	FA	5	6	Acetyl-CoA, Oxaloacetate	−1	0	−1	−1	0

**Table 3:** ATP and co-factors produced from glucose, palmitate, serine, and glutamine when RedOx balance is the main metabolic constraint. Glucose contributes to the generation of NADPH through the oxidative and reductive cycles of pentose phosphate pathway (PPP). The number of reactions is calculated assuming the smallest number needed to generate the most amount of NADPH. Serine catabolism through mitochondrial one carbon cycle can generate NADPH as a side product of the enzymatic activity of MTHFD2/L and ALDH1L2. Palmitate oxidation is shown here as an example for the potential of generating reducing power in the form of NADPH via NNT activity. Moreover, all mitochondrial NADH molecules generated in the other examples are assumed to be converted to NADPH. Conversion of malate to pyruvate via ME1 can generate NADPH instead of NADH during glutamine catabolism [145,146,147]. 1C, one-carbon; NNT, nicotinamide nucleotide transhydrogenase; ME, malic enzyme; RXN, reaction; C, carbons per substrate molecule.

Substrate	Pathway	C	RXNs	Products	ATP	NADPH	FADH_2_	Total ATP	NADPH/RXN
Glucose	Oxidative & Reductive PPP	6	49	6 CO_2_	−1	12	0	0	0.24
Serine	1C Cycle	3	4	Gly, CO_2_	0	2	0	0	0.5
Palmitate	Potential Action of NNT	16	101	16 CO_2_	6	31	15	28.5	0.31
Glutamine	TCA Cycle, ME1	5	8	Lactate, 2 CO_2_	1	2	1	2.5	0.25
